# In Vivo Evaluation of the Chronic Oral Toxicity of the Marine Toxin Palytoxin

**DOI:** 10.3390/toxins12080489

**Published:** 2020-07-30

**Authors:** Andrea Boente-Juncal, Sandra Raposo-García, Carmen Vale, M. Carmen Louzao, Paz Otero, Luis M. Botana

**Affiliations:** Departamento de Farmacoloxía, Farmacia e Tecnoloxía Farmacéutica, Facultade de Veterinaria, Universidade de Santiago de Compostela, Campus Universitario s/n, 27002 Lugo, Spain; andrea.boente.juncal@usc.es (A.B.-J.); sandra.raposo@rai.usc.es (S.R.-G.); mcarmen.louzao@usc.es (M.C.L.); paz.otero@usc.es (P.O.)

**Keywords:** palytoxin, marine toxins, in vivo toxicity, EFSA, risk assessment, sodium-potassium ATPase, LD_50_, NOAEL

## Abstract

*Palytoxin* (PLTX) is one of the most poisonous substances known to date and considered as an emergent toxin in Europe. Palytoxin binds to the Na^+^-K^+^ ATPase, converting the enzyme in a permeant cation channel. This toxin is known for causing human fatal intoxications associated with the consumption of contaminated fish and crustaceans such as crabs, groupers, mackerel, and parrotfish. Human intoxications by PLTX after consumption of contaminated fishery products are a serious health issue and can be fatal. Different reports have previously explored the acute oral toxicity of PLTX in mice. Although the presence of palytoxin in marine products is currently not regulated in Europe, the European Food Safety Authority expressed its opinion on PLTX and demanded assessment for chronic toxicity studies of this potent marine toxin. In this study, the chronic toxicity of palytoxin was evaluated after oral administration to mice by gavage during a 28-day period. After chronic exposure of mice to the toxin, a lethal dose 50 (LD_50_) of 0.44 µg/kg of PLTX and a No-Observed-Adverse-Effect Level (NOAEL) of 0.03 µg/kg for repeated daily oral administration of PLTX were determined. These results indicate a much higher chronic toxicity of PLTX and a lower NOAEL than that previously described in shorter treatment periods, pointing out the need to further reevaluate the levels of this compound in marine products.

## 1. Introduction

The marine toxin palytoxin (PLTX) is one of the most toxic marine phycotoxins known to date. Since its first isolation, about 50 years ago, from marine corals of the genus *Palythoa* [1] and later on from algae of the genus *Ostreopsis* [2,3,4], the toxin was associated with high toxicity. Even though the toxic organisms producers of PLTX were initially located in the Pacific Sea, now the presence of the toxin and its congeners has expanded also to European waters. In fact, a recent report described a massive bloom of the dinoflagellate *Ostreopsis* cf. *ovata* producer of 5.6 pg of PLTX per cell in the Adriatic Sea [5]. This bloom was not an isolated case but rather massive *Ostreopsi*s blooms have been reported in the Mediterranean Sea during the last 20 years as summarized in several reports [5] and increasingly reported in the literature [2,3,5,6,7,8,9,10], and are in expansion mainly due to environmental factors [11,12,13].

The increment in toxic blooms of PLTX in Europe raised high concern and the urgent need to obtain toxicity data useful for the future regulation of this toxin in marine products captured in European coasts. Besides causing human intoxications, after consumption of marine crustaceans and fish [14,15,16,17], toxic effects of different severity have been attributed to PLTX after human inhalation of marine aerosols or skin contact during *Ostreopsis* blooms [18,19,20] and even after dermal or inhalatory exposition to zoanthid corals in aquariums [21,22].

The mechanism of action of palytoxin is well established because the toxin transforms the sodium-potassium pump (Na^+^/K^+^-ATPase), an enzyme crucial to maintain the sodium and potassium gradients across all the cell membranes, in a non-selective cation channel [23,24,25], which leads to the increase in intracellular potassium levels [26,27]. The sodium-potassium pump is ubiquitously distributed in different cells types and the interaction of PLTX with the enzyme has been studied in a wide variety of in vitro [28,29,30,31,32,33,34] and in vivo systems [29,35,36,37,38]; therefore, the benthic microalgal proliferation of the dinoflagellates producers of PLTX and its analogues causes respiratory, muscular, cardiovascular, gastrointestinal, and nervous alterations in humans [39]. After ingestion of PLTX-contaminated fishery products, several fatal human intoxications with symptomatology characterized by gastrointestinal alterations, myalgia and spasms, have been reported [38,40]. Initial studies on the chronic PLTX toxicity after repeated daily oral administration of PLTX to mice allowed to determine a No-Observed-Adverse-Effect Level (NOAEL) of 3 µg/kg/day for a 7-day exposure period [28]. Moreover, we have recently shown that a single oral dose of 15 µg/kg PLTX elicited statistically significant subtle changes in mouse LDH plasma levels after a 96 h observation period [35].

Besides their high toxicity, currently, neither PLTX nor its analogues are regulated in fishery products in Europe or in other countries, but the European Food Safety Authority (EFSA) has recommended a maximum of 30 µg PLTX and analogues/kg of shellfish meat [41], and in the same reports, an oral acute reference dose (ARfD) of 0.2 µg/kg body weight (BW) for the total content of PLTX and its analogue Ostreocin-D was derived. However, this report remarks the lack of chronic toxicity data for this group of toxins. In fact, while several reports have evaluated the acute toxicity of PLTX by several administration routes [36,37,42], the acute oral toxicity of this group of toxins increases with longer times of observation. Therefore, in this work we aimed to evaluate the chronic toxicity of PLTX following the principles of the internationally adopted Organisation for Economic Co-operation and Development (OECD) guidelines for the 28 days exposure period, as adapted to comply with the principle of reduction required by the Directive 2010/63/EU [43]. In this report, Swiss mice were fed daily with PLTX, by gavage, at doses of 0.03, 0.1, 0.3, 1, 3.5, and 10 µg/kg and animals were monitored for the corresponding survival periods ending the experiments after 28 days of PLTX administration. The data presented here indicate that daily chronic exposure of mice to the toxin has profound effects on blood biochemistry and urine parameters. In addition, daily dosage of mice with PLTX elicited macroscopic alterations in the digestive tract and ultrastructural alterations in the stomach.

## 2. Results

### 2.1. Chronic Toxicity Elicited by Daily Oral PLTX

Based on the initial results for the acute toxicity of PLTX in mice [35], we further explored the chronic toxicity of this compound assessing its oral toxicity after a 28 daily exposure period. Thus, the first dose of palytoxin evaluated to test its chronic toxicity was 3.5 µg/kg/day over a 28 day treatment period, but with this dose a mortality of about 75% was observed, therefore, subsequent dilutions of the toxin were made in order to achieve the chronic NOAEL for the toxin. Table 1 represents the mortality rate at the different PLTX doses evaluated. As shown in this table, at 3.5 µg/kg/day, only two out of eight mice survived the 28-day treatment period. At the highest dose evaluated (10 µg/kg/day) animals survived 8, 10 and 18 days and none of the animals evaluated completed the entire treatment period.

**Figure 1:**
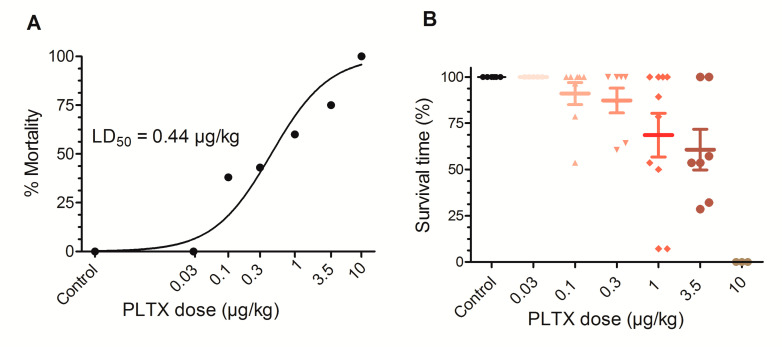
Chronic oral LD_50_ for PLTX (**A**) and survival times for each mouse treated with the different PLTX doses (**B**). Scatter plot graph showing the survival time for each animal. Values are means ± SEM of the data obtained from three to ten animals.

As shown in Figure 1A, non-linear fit of the data represented on Table 1 allowed to calculate a chronic lethal dose 50 (LD_50_) for PLTX of 0.44 µg/kg (95% confidence interval (CI) from 0.22 to 0.9 µg/kg, *R*^2^ = 0.9122, df (degrees of freedom) = 7), although the survival times differed considerably between the individual mice fed with each dose of PLTX as depicted in Figure 1B.

All over the treatment period, mice weight was monitored weekly. As shown in Table 2, control mice and mice treated with PLTX at doses of 0.03, 0.1 and 0.3 µg/kg gained some weight during the 28 days treatment period while mice dosed with PLTX at 1 and 3.5 µg/kg lost weight during the treatment. Average weight of the animals during the treatment period and accumulative weekly weight change are shown in Table 2. Mice dosed with PLTX at 1 and 3.5 µg/kg exhibited significant weight lost at day 7 and day 14 of treatment and mice treated with 10 µg/kg lost weight at day 14, but this data was not significantly different due to the limited number of animals.

Even at PLTX doses as low as 0.1 µg/kg, animals fed with the toxin administered daily by gavage exhibited symptoms typical of the toxin that are summarized in Table 3, specifying the number of animals that exhibited the different symptoms. No symptoms were observed after dosing the mice with 0.03 µg/kg PLTX, while piloerection was the most frequent observation at the dose of 0.1 µg/kg PLTX. At the 0.3 µg/kg PLTX dose, the most common symptoms were lethargy, piloerection, and facial swelling. In addition, dyspnea appeared at the doses of 1, 3.5 and 10 µg/kg together with signs of abdominal pain and, as in our preliminary acute study [35], kyphosis and ataxia were also present after PLTX administration.

### 2.2. Chronic Oral PLTX Induced Macroscopic Changes in the Intestines and Stomachs of Treated Animals

**Figure 2:**
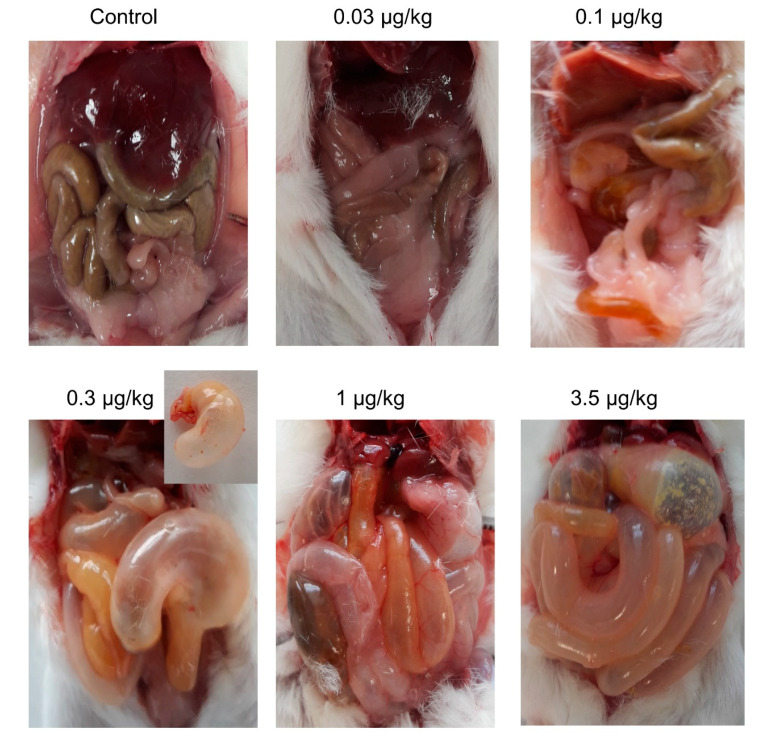
Representative image of the organs from control mice or mice treated daily with PLTX at doses of 0.03, 0.1 (survival time 28 days), 0.3 (survival time 18 days), 1 (survival time 25 days), or 3.5 µg/kg (survival time 15 days).

After euthanasia, the animals were subjected to macroscopic observation on necropsy. As shown in Figure 2, no macroscopic alterations were observed neither in control animals nor in animals fed 28 days with 0.03 µg/kg PLTX. However, in one animal treated with 0.1 µg/kg PLTX that survived for 27 days, the stomach and small intestines were empty of solid contents and instead these structures contained dark-yellow mucous liquid. At the same dose, after daily feeding with 0.1 µg/kg PLTX, two mice showed swollen abdomen at death and survival times of 15 and 27 days. At the dose of 0.3 µg/kg, one mouse that survived 18 days of treatment showed gas accumulation in the stomach and intestines and absence of solid content. For this group of mice, two out of seven animals presented abdominal swelling and survival times of 18 and 24 days. Similarly, stomach and intestine alterations were observed in other mice fed daily with 1 µg/kg PLTX and that survived 25 days. At this dose, 4 out of 10 animals showed abdominal swelling and survival times of 2, 15, 25, and 28 days from the beginning of treatment. Similar alterations were observed in a mouse fed daily with 3.5 µg/kg PLTX that survived for 15 days. At this dose, five out of eight mice showed abdominal swelling at death (survival times ranged from 7 h for one animal, 9 days for another mouse, 15 days for 2 mice, and 28 days in the last animal with abdominal swelling). Notwithstanding, it is noteworthy to remark that abdominal swelling and the presence of gas and mucus in the stomach and intestines of PLTX-treated animals was a common feature. At the highest dose employed, 10 µg/kg, only one mouse, which survived 8 days, did not present changes at necropsy.

### 2.3. Chronic Oral PLTX at Doses of 1 and 3.5 µg/kg Decreased Urine Production After the 28 Days Treatment Period

On day 27 after the beginning of treatment, mice were housed individually in metabolic cages and the last dose of toxin was administered by gavage, before monitoring food consumption and urine and faeces production over a 24 h period. As shown in Table 4, at the highest doses evaluated after daily oral administration of PLTX, urine production decreased by about 88% at the doses of 1 and 3.5 µg/kg (Table 4). Urine samples were limited to the number of animals that survived the entire treatment.

### 2.4. Urine Parameters in Control Mice and in Mice Treated Daily with PLTX

Urine parameters were evaluated in samples collected during the 24 h period before sacrifice, after mouse placement in individual metabolic cages. The animals employed for this analysis were the mice that survived the 28 days treatment period. Urine parameters analyzed included colour, clarity, specific gravity, protein, glucose, ketones, blood/hemoglobin, bilirubin, and urobilinogen. Urine parameters in control and PLTX-treated mice are summarized in Table 5. No statistically significant differences were found between control mice and animals dosed daily with PLTX besides a bilirubin increase at PLTX doses of 0.1 µg/kg (*t* = 2.959; df = 9; *p* = 0.016) and 3.5 µg/kg (*t* = 2.579; df = 6; *p* = 0.0418).

### 2.5. Effect of 28 Day Repeated Exposure of Mice to Low Doses of PLTX on Blood Biochemical Parameters

**Figure 3:**
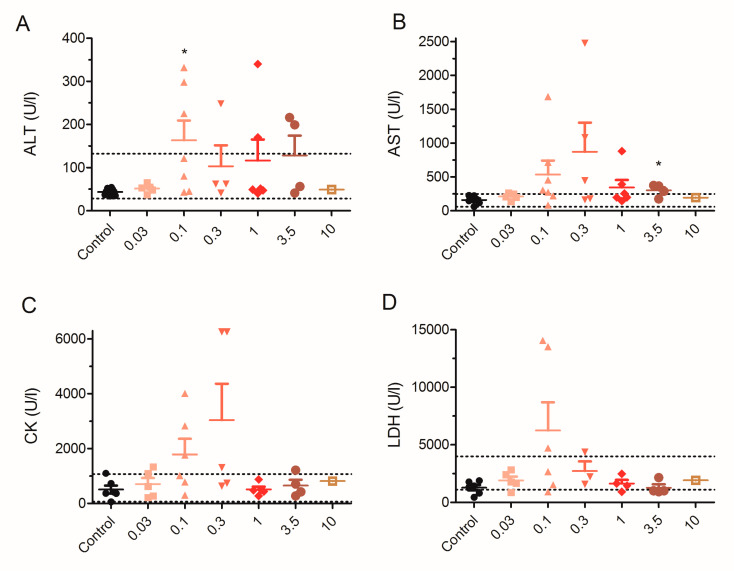
Scatter plot graphs showing the effects of different doses of PLTX administered daily by gavage on the blood levels of ALT (**A**), AST (**B**), CK (**C**), and LDH (**D**). Data are expressed as means ± SEM from one to seven determinations. * *p* < 0.05 versus control mice. The respective minimum and maximum reference blood values for each parameter are marked by the pointed lines.

At the end of the 28 days of treatment, mice were euthanized, and biochemical analysis was performed in blood extracted by cardiac puncture immediately after euthanasia or, in some cases, after sudden death of animals. Blood samples were analyzed as in the previous study [35] and blood parameters analyzed included electrolyte levels (Cl^−^, Na^+^ and K^+^), alanine transaminase (ALT), aspartate transaminase (AST), lactate dehydrogenase (LDH), and creatine kinase (CK). As shown in Figure 3A, mean blood levels of ALT in control animals were 44 ± 4 U/L (*n* = 6) and 52 ± 5 U/L (*n* = 5) in animals dosed with 0.03 µg/kg. ALT blood levels were significantly increased to 163 ± 46 U/L in seven animals dosed with 0.1 µg/kg PLTX (*t* = 2.410; df = 11; *p* = 0.0346). In mice fed orally with PLTX at 0.3 µg/kg ALT blood levels were 103 ± 49 U/L, 116 ± 49 U/L in mice fed with 1 µg/kg PLTX, 128 ± 46 U/L in the four mice fed with 3.5 µg/kg PLTX, and 49 U/L in one mice that survived 10 days after daily PLTX dosage. Besides mean AST blood levels (Figure 3B), were 157 ± 26 U/L (*n* = 6) in control mice, 210 ± 23 U/L in mice dosed daily with 0.03 µg/kg (*n* = 5), 535 ± 206 U/L (*n* = 7) in mice treated with 0.1 µg/kg PLTX, 869 ± 435 U/L (*n* = 5) in mice fed with 0.3 µg/kg PLTX, 343 ± 113 U/L (*n* = 6) in mice fed orally with PLTX at 1 µg/kg, 301 ± 47 U/L (*n* = 4) in mice fed with 3.5 µg/kg, and 194 U/L in one mice after 10 days administration of PLTX at 10 µg/kg. Statistical analysis of AST data did not yield significant differences between the control group and the PLTX-treated animals with the exception of mice fed with 3.5 µg/kg (*t* = 2.935; df = 8; *p* = 0.0189), but the levels of AST in 0.1, 0.3, 1 and 3.5 groups were above the normal reference range (59–247 U/L for the IDEXX). In contrast, creatine kinase blood levels (Figure 3C) were 508 ± 147 U/L for the control group (*n* = 6), 708 ± 220 U/L for the group of mice dosed with 0.03 µg/kg (*n* = 5), 1781 ± 572 U/L (*n* = 6), 3040 ± 1319 U/L (*n* = 5), 512 ± 99 U/L (*n* = 5), 654 ± 208 U/L (*n* = 4), and 814 U/L (*n* = 1) for the groups of mice dosed with 0.1, 0.3, 1, 3.5 and 10 µg/kg PLTX, respectively. Statistical comparison of creatine kinase data did not show significant differences between control animals and animals treated daily with PLTX. Nevertheless, in this case, the blood level of CK in the animals dosed daily with 0.1 and 0.3 µg/kg of PLTX was well above the IDEXX reference range, which is between 68 and 1070 U/L, with some animals showing CK levels well above 1070 U/L. As shown in Figure 3D, mean LDH values were 1277 ± 229 U/L (*n* = 6) in the control group, 1904 ± 338 U/L in three animals dosed with 0.03 µg/kg, 6230 ± 2448 U/L (*n* = 6) in the 0.1 µg/kg group, 2716 ± 841 U/L (*n* = 3) for the 0.3 µg/kg group, 1626 ± 325 U/L (*n* = 4) in the 1 µg/kg group, 1275 ± 296 U/L (*n* = 4) in the 3.5 µg/kg group, and finally 1914 U/L in one mouse at the highest dose employed. In this case, no statistically significant differences were observed between control animals and those that received a daily PLTX dose. However, two out of the six mice fed with 0.1 µg/kg PLTX had LDH levels well above the IDEXX reference range for blood LDH, which is between 1105 and 3993 U/L.

**Figure 4:**
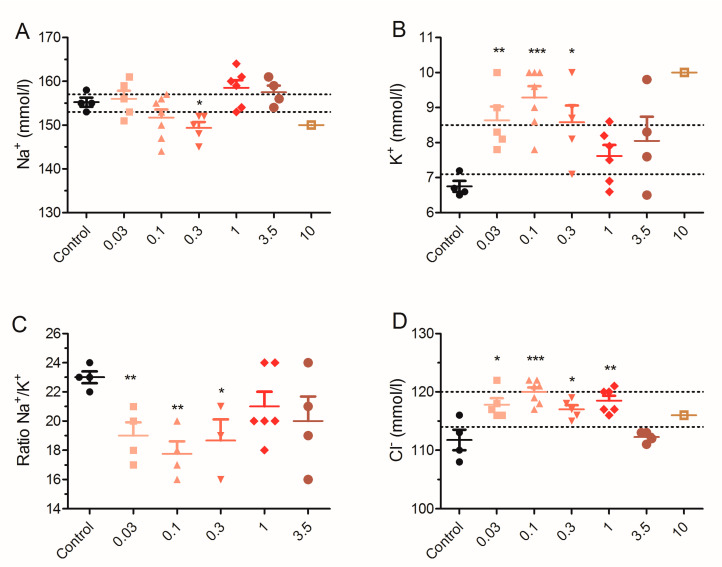
Scatter plot representation of the blood levels of sodium (**A**), potassium (**B**), ratio sodium/potassium (**C**) and chloride (**D**) in control Swiss female mice and in mice dosed daily by gavage with PLTX at 0.03, 0.1, 0.3, 1, 3.5, and 10 µg/kg. * *p* < 0.05, ** *p* < 0.01, *** *p* < 0.001 versus control mice. The respective minimum and maximum reference values of blood electrolyte levels are indicated by the pointed lines.

Next, the effect of repeated exposure of mice to low doses of PLTX on blood electrolyte levels was evaluated (Figure 4). Statistically significant differences were found in sodium and potassium levels as well as in the Na^+^/K^+^ ratio and on blood chloride levels, this dysregulation of electrolytes levels is in agreement with the previously reported alterations produced by a single oral dose of PLTX [35].

As shown in Figure 4A, mean Na^+^ blood values in control animals were 155 ± 1 mmol/L (*n* = 4) while in PLTX-treated animals, sodium levels were 156 ± 2 mmol/L (*n* = 5) at 0.03 µg/kg, 152 ± 2 mmol/L in seven animals dosed with 0.1 µg/kg of PLTX. At the dose of 0.3 µg/kg of PLTX, five animals have mean blood Na^+^ values of 149 ± 1 mmol/L and significant differences were observed versus control animals (*t* = 3.332, df = 7, *p* = 0.0126). Similarly, blood sodium levels were 159 ± 2 mmol/L (*n* = 6) and 158 ± 2 mmol/L (*n* = 4) in animals dosed with 1 and 3.5 µg/kg PLTX, respectively. Moreover, at the highest dose employed (10 µg/kg), the blood sodium level was 150 mmol/L, but only one mouse was evaluated. Interestingly, an increase in potassium levels was found in blood samples as indicated in Figure 4B. Animals fed daily with PLTX doses of 0.03, 0.1, and 0.3 µg/kg presented a statistically significant increase in blood potassium levels comparing with control animals. In control animals, K^+^ levels were 6.8 ± 0.2 mmol/L (*n* = 4), 8.6 ± 0.1 mmol/L (*n* = 5) in mice fed daily with 0.03 µg/kg PLTX (*t* = 4.053; df = 7; *p* = 0.0048), 9.3 ± 0.3 mmol/L in seven mice fed with 0.1 µg/kg PLTX (*t* = 5.611; df = 9; *p* = 0.0003) and 8.6 ± 0.5 mmol/L (*n* = 5) in mice treated with 0.3 µg/kg PLTX (*t* = 3.255; df = 7; *p* = 0.0140). Despite the short survival time of the animals treated with the highest PLTX dose, blood K^+^ levels were well above the normal range in one mouse that survived 10 days of treatment. Due to the decrease in blood sodium concentration and the increase in blood potassium levels, the ratio Na^+^/K^+^ decreased (Figure 4C), especially at low doses of PLTX, for which survival times were longer. In control mice, the ratio Na^+^/K^+^ was 23 (*n* = 4) and it decreased to 19 ± 1 (*t* = 4.000, df = 6, *p* = 0.0071), 18 ± 1 (*t* = 5.547, df = 6, *p* = 0.0015) and 19 ± 1 (*t* = 3.313, df = 5, *p* = 0.0212) in animals treated with 0.03, 0.1 and 0.3 µg/kg PLTX, respectively. Next, the effects of oral PLTX on blood chloride levels were evaluated (Figure 4D). Chloride blood levels increased compared with control, and statistically significant variations were found between control animals and PLTX-treated animals. Thus, Cl^−^ levels were 112 ± 2 mmol/L in four control animals, 118 ± 1 mmol/L (*t* = 3.041, df = 7, *p* = 0.0188) in animals fed daily with 0.03 µg/kg PLTX, 120 ± 1 mmol/L (*t* = 5.066, df = 9, *p* = 0.0007) after the 0.1 µg/kg treatment, 117 ± 1 mmol/L (*p* = 0.0192) after the 0.3 µg/kg treatment, and 119 ± 1 mmol/L (*p* = 0.0047) after the 1 µg/kg treatment.

### 2.6. Ultrastructural Examination of Stomach Damage Induced by Repeated Oral Dosing of Mice with PLTX

**Figure 5:**
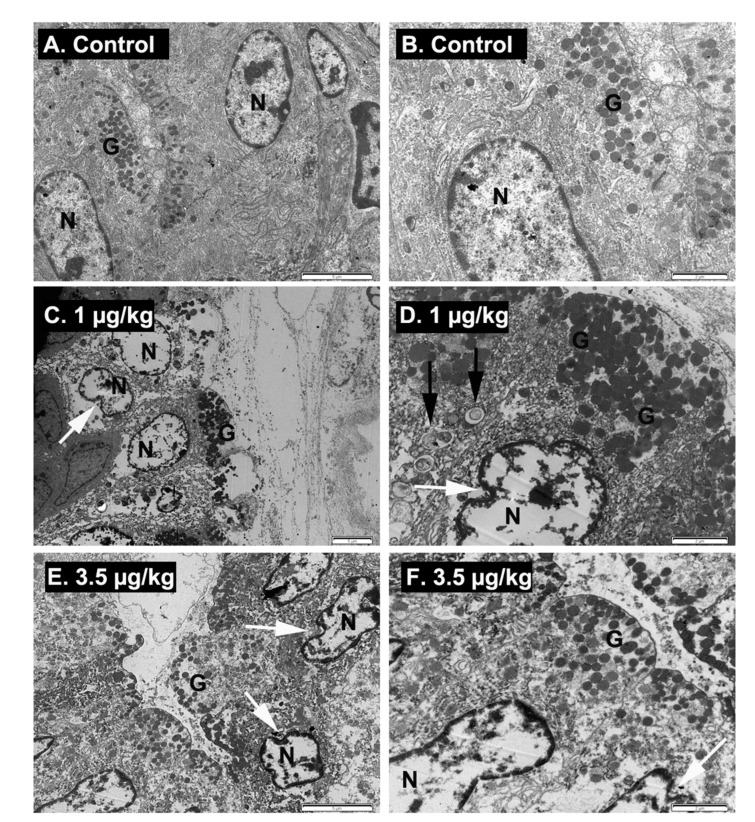
TEM micrographs showing mucous cells of the stomach from control mice (**A**,**B**), from mice treated with PLTX at 1 µg/kg during 9 days (**C**,**D**), and from mice treated daily with PLTX at 3.5 µg/kg during 22 days (**E**,**F**). Different magnifications are shown; scale bars are 5 µm (left panel) and 2 µm (right panel) respectively. N: nucleus, G: electron-lucent granules of mucous cells. Evident deformation of nuclei (white arrows), and cytoplasm disintegration were observed in the mucous cells of PLTX- treated mice with autophagosomes (black arrows).

Since, after necropsy, macroscopic changes in the digestive tract of PLTX-treated animals were observed, and some mice dosed with PLTX at 1 and 3.5 µg/kg exhibited significant weight loss, transmission electron microscopic studies (TEM) of the stomach were performed. Samples from control animals and from animals treated with PLTX were obtained immediately after euthanasia and evaluated. First, and in view of the macroscopic alterations of the stomach with dark-yellow liquid and dilatation with gas accumulation, mucous cells were evaluated. After TEM analysis, some evident changes in the structure of mucous cells, which were identified by their electron-lucent granules, were appreciated as Figure 5 shows. Thus, in control animals, a regular structure of the mucous cells was observed (Figure 5A,B), while in animals treated daily with PLTX at doses of 1 µg/kg during 9 days (Figure 5C,D) or 3.5 µg/kg for 22 days (Figure 5E,F), the surface of mucous cells was damaged and most mucous cells showed an irregular nuclei with chromatin margination and vacuolated cytoplasm with numerous autophagosomes.

**Figure 6:**
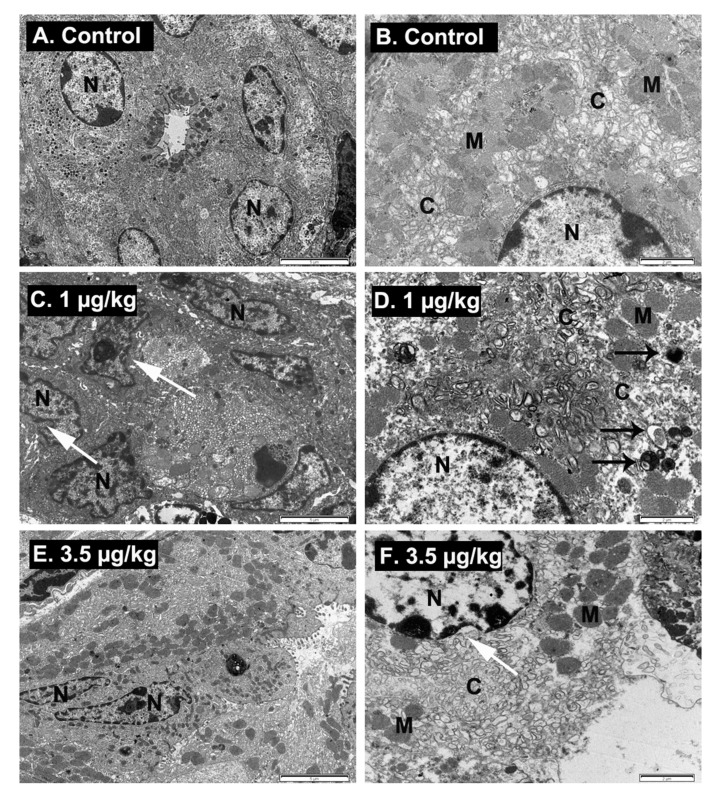
Representative electron micrographs depicting parietal cells of control animals (**A**,**B**) and of animals treated daily with 1 µg/kg PLTX (**C**,**D**) or PLTX at 3.5 µg/kg (**E**,**F**). Scale bar is 5 μm, and 2 μm, respectively from the left to the right panels. N: nucleus, C: canalicular system, M: mitochondria. Animals fed with PLTX showed apoptotic nuclei with nucleus membrane shrinkage (white arrows), abundant autophagosomes (black arrows), electron dense mitochondria and dilatation of the oval vesicles of the canalicular system with evident wide clear centers.

Aside from mucous cells, the gastric epithelium secretes hydrochloric acid and digestive enzymes, and since palytoxin altered blood ionic homeostasis, the acid-producing cells of the stomach (parietal cells) were also analyzed by TEM. As shown in Figure 6, in control animals, parietal cells conserved their characteristic microvilli, membrane invaginations called canaliculi and clusters of round vesicles that transfer substances from the cytoplasm to the lumen of the canalicular system (Figure 6A,B). In contrast, in animals fed daily with PLTX at the dose of 1 µg/kg (Figure 6C,D), evident alterations of the parietal cells were observed, consisting of damage of the cytoplasm with dilatation of the canaliculi system, electron dense mitochondria and proliferation of autophagosomes (black arrows). Moreover, in PLTX-treated animals, nuclear membrane shrinkage, chromatin margination, and development of membrane-bound remnants of the nuclear membrane also called apoptotic bodies were observed. These alterations are consistent with programmed cell death that leads to apoptosis of the parietal cells of PLTX-treated animals [44]. The same ultrastructural alterations were observed in mice dosed daily with 3.5 µg/kg of PLTX (Figure 6E,F).

## 3. Discussion

So far, the available in vivo toxicity studies with palytoxin have shown a high acute oral toxicity with LD_50_ ranging from 510 to 767 µg/kg. Nevertheless, in the latest opinion of the EFSA experts on PLTX-related risks [41], the lack of data on the subchronic and chronic toxicity of PLTX and its analogues was highlighted. In view of these requirements, in this work we pursued to determine the chronic LD_50_ and the chronic NOAEL for oral PLTX. In this context, the data presented in this report constitute the first initial approach for the chronic evaluation of the PLTX toxicity after daily ingestion. Moreover, as far as we know, this is the first work that evaluates the chronic toxicity of this potent marine toxin following the OECD recommendations to evaluate the chronic toxicity of chemicals [43]. The data presented here indicate that daily feeding of female Swiss mice by gavage with different PLTX doses for 28 days resulted in an oral LD_50_ of 0.44 µg/kg and a NOAEL of 0.03 µg/kg. In view of the high increase of toxicity after repeated exposure to PLTX, it is crucial to consider a representative scenario in which successive exposures to low doses of PLTX might arise, especially for the population living near coastal areas affected by *Ostreopsis* blooms. Indeed, besides oral toxicity, many reports have described toxic corneal reactions [45,46], as well as frequent respiratory and dermal alterations in humans [18,20,47,48].

Until now, different in vivo toxicities for PLTX in mice have been reported, depending mainly on the time of exposure of the animals to the toxin. For instance, a NOAEL of 300 µg/kg and a LD_50_ of 757 µg/kg have been reported after a single dose of PLTX and an observation period of 24 h [37]. In the same conditions, an LD_50_ of 510 µg/kg was reported by other authors [49], while no death was observed in mice after oral administration of PLTX at doses up to 200 or 300 µg/kg [42] but stomach alterations were evident 2 h after toxin administration. However, the toxin lethality increased in mice after oral repeated administration of successive doses and this was associated to a decrease in the NOAEL up to 3 µg/kg after a repeated oral dose of PLTX for 7 days [28]. However, to date, no additional reports on the chronic toxicity of PLTX have been released. We demonstrated here that after daily feeding of mice with PLTX during 28 days, a daily oral dose of 3.5 µg/kg led to a mortality rate of about 75% with survival times between 7 h and 28 days. In this context, it is important to remark that the NOAEL was much lower than that previously reported, since even after oral administration of the toxin at doses of 1 µg/kg, animals exhibited stomach ultrastructural alterations after 9 days of treatment and characteristic symptoms, some of them initiating on the third day of treatment. The results presented here indicate that after the 28-day treatment period, mice dosed with 0.03 μg/kg of PLTX did not present changes in most of the blood parameters analyzed in this work. However, the only alteration observed in mice fed with 0.03 µg/kg PLTX was hyperkalemia that appeared in two out of five animals. However, the modifications in blood electrolyte levels were much lower at this dose than that observed at higher toxin concentrations. These results are in consonance with previous studies that reported electrolyte changes after PLTX administration [28,35,38].

As the molecular target of PLTX, the Na^+^-K^+^-ATPase pump is indispensable for different cellular functions, and its inhibition results in an excess of extracellular K^+^ and accumulation of intracellular Na^+^ because, in the presence of toxin the Na^+^-K^+^- ATPase can no longer pump K^+^ into the cell or pump Na^+^ out of the cell [50], the resulting blood electrolyte homeostasis dysregulation observed in animals fed daily with PLTX was expected. The pathophysiology expected after PLTX intoxication, includes cardiovascular alterations as a consequence of the Na^+^-K^+^ inhibition [51] and could result in other clinical signs such as piloerection, dyspnea, and abdominal pain that were even present in some mice fed daily with 0.1 µg/kg of PLTX. Clinical signs including lethargy, dyspnea and consequently cyanosis in the tail of the animals and neuromuscular alterations, kyphosis, circling, and ataxia were also present after daily PLTX administration, in consonance with the symptoms reported in other in vivo studies using higher doses of PLTX and shorter exposure periods [28,29,35,38]. Additionally, macroscopic alterations of the digestive tract were also present in animals fed with PLTX at doses of 0.3 µg/kg and higher. This observation is in agreement with previous data indicating gastric lesions after acute oral administration of higher PLTX doses [35,38,42]. Regarding electron microscopy analysis of the stomach, the results presented here showed a strong ultrastructural damage of the mucous and parietal cells which could probably lead to erosion and detachment of the gastric epithelium. This fact could be related with earlier PLTX studies reporting that the Na^+^-K^+^-ATPase was transformed in an open cation channel in the presence of PLTX [23,25] which will lead to cell membrane depolarization and the consequent change in ion channel conductance. Thus, it was expected that the sustained overload of the Na^+^ concentration would affect all the ion transport systems coupled to the sodium gradient like the Na^+^/H^+^ exchanger, the Na^+^/Ca^2+^ exchanger, the H^+^-K^+^-ATPase pump and hence, the osmotic balance [52,53]. In this regard, death of parietal cells has been observed after inhibition of the H^+^-K^+^-ATPase with omeprazol [54,55]. In addition to the gastric alterations observed by electron microscopy, it is noteworthy to point out that blood electrolytes imbalances together with gastric acid changes could lead to nutrient deficiency and consequently weight loss [56], as observed after daily administration of PLTX at doses of 1 and 3.5 μg/kg.

Altogether, the new findings described in the present study, together with the increase in *Ostreopsis* blooms in Europe, raise the need to perform additional studies on the oral in vivo toxicity of this potent marine toxin in order to prevent human illness and accurately regulate the food levels of this emerging marine toxin.

## 4. Conclusions

Daily feeding of female Swiss mice by gavage with different PLTX doses for 28 days resulted in an oral LD_50_ of 0.44 µg/kg and a NOAEL of 0.03 µg/kg.

## 5. Materials and Methods

### 5.1. In Vivo Experimental Procedure

In vivo studies were performed with Swiss female mice weighing an average of 21 to 25 g in each group (4 weeks old). All in vivo studies employed in the manuscript followed the European directives (EU directive 2010/63/EU) and the Spanish legislation (Real Decreto 53/2013, Decreto 296/2008), and were performed accordingly with the principles approved by the Institutional Animal Care Committee of the Universidade de Santiago de Compostela under procedure number 011/14, authorized on 14 August 2014 (MR110250) and under procedure number 06/19/LU-002, authorized on 1 March 2019 (A12X00509).

The PLTX used in the experiments was purchased from Fujifilm Wako Pure Chemical Corporation (Osaka, Japan) extracted from *Palythoa tuberculosa*, (purity 88.9% and CAS Number: 77734-91-9) and dissolved in water. Before administration, the stock PLTX solution was diluted in 0.9% saline solution to achieve each dose. For the treatment, mice received a daily oral dose of PLTX by gavage (200 µL of physiological saline at 9:30 am) or the same volume of control saline. Animals were observed closely for the first 2 h and periodically thereafter, until sacrifice of surviving animals at the end of the treatments. During the course of the experiments, moribund animals or animals obviously in pain or showing signs of agony, as well as animals with weight loss higher than 10% and enduring distress, were euthanized and considered in the interpretation of the test results in the same way as animals that were euthanized at the end of the 28 days treatment period. During treatment, mice were housed in rooms with constant temperature, humidity, and a controlled photoperiod at the animal facilities of the School of Veterinary Medicine of the University of Santiago de Compostela (Code: AE-LU-002). During experiments, control and PLTX-treated mice were weighted weekly at day 0 (day of the fist treatment), day 7, day 14, day 21 and day 28, and food consumption was also registered at the same intervals. Euthanasia was performed on day 28. After the last PLTX-dosage (day 27), control and PLTX-treated mice were placed in metabolic cages for 24 h to monitor urine and faeces production and obtain samples. Finally, animals were euthanized in a CO_2_ chamber, and immediately after death confirmation by cervical dislocation, blood and tissue samples were collected. All animals in the study were subjected to a full necropsy, including detailed visualization of heart, liver, lungs, kidneys, spleen, stomach, duodenum, rectum, and cerebrum.

### 5.2. Evaluation of Enzyme and Electrolyte Levels in Plasma of Control and PLTX-Treated Mice

Blood was obtained directly from the ventricle of euthanized mice as previously described [35,57] and conserved in microtubes containing lithium heparin (2.5 U.I./mL) until the end of the necropsy and tissue collection (30 min). Afterwards, each blood sample was mixed for 30 s and centrifugated for 90 s at 15,800 rpm/12,000× *g* (IDEXX Stat Spin, IDEXX Europe B.V., Hoofddorp, The Netherlands). The computer controlled biochemical analyzer IDEXX Catalyst Dx (IDEXX VetLab Station, IDEXX Europe B.V) was used for the analysis of plasma parameters. In most of the samples, 300 µL of plasma were used to evaluate electrolyte levels (Cl^−^, Na^+^ and K^+^), alanine transaminase, aspartate transaminase, lactate dehydrogenase, and creatine kinase. Initially, all the markers were evaluated in undiluted samples, but if the marker level in plasma was above the reference range of the analyzer, an automatic dilution with physiological saline was performed. Samples with altered visual properties that could lead to the misinterpretation of the biochemical parameters (high hemolysis, icterus or lipidemia) were not further processed.

### 5.3. Urine Analysis

Urine colour, clarity, protein, glucose, ketones, blood hemoglobin, bilirubin, and urobilinogen analysis were performed using a reflectance photometer (IDEXX VetLab UA), which reads and evaluates IDEXX UA strips. Urine specific gravity was measured with a refractometer. Because for some mice a small amount of blood or urine were obtained, it was not possible to measure all the parameters for each animal.

### 5.4. Ultraestructural Analysis

Stomach samples preparation for transmission electron microscopy was performed according to previously described procedures [35,57,58]. Concisely, organ samples (1 mm^3^) were fixed by immersion (2.5% glutaraldehyde in phosphate buffered saline (PBS) containing in mM: 137 NaCl, 8.2 Na_2_HPO_4_, 1.5 KH_2_PO_4_ and 3.2 KCl) for 2 h at 4 °C and rinsed three times with PBS. Postfixation by immersion in 1% OsO_4_ in 0.1 M cacodylate trihydrate buffer was performed for 60 min. After, tissues were rinsed and dehydrated in graded ethanol solutions, including one bath with 70% ethanol and 0.5% uranyl acetate, rinsed in propylene oxide, and embedded in Epon 812 (Momentive Specialty Chemicals Inc., Houston, TX, USA). A Leica Ultracut UCT ultramicrotome (Leica Microsystems GmbH, Wetzlar, Germany) was used to obtain ultrathin sections of the tissue samples. Samples were counterstained with uranyl acetate and lead citrate, and ultrastructural analysis of 1 mm^2^ thick samples was performed with a JEOL JEM-1011 Transmission Electron Microscope (Jeol Ltd., Tokyo, Japan).

### 5.5. Statistical Analysis

Statistical analyses were carried out using GraphPad Prism 5 (GraphPad Software, Inc, La Jolla, CA, USA). All values are expressed as mean ± SEM. A *p*-value of < 0.05 was considered statistically significant. ANOVA repeated measures followed by the Dunnett’s comparison test was used for statistical comparisons.

## Figures and Tables

**Table 1:** Percent of mortality and survival times observed for each mouse fed daily with PLTX during a 28 day period.

Dose (µg/kg)	Total Mice	Dead Mice	Individual Survival Time (Days) (Mean Survival Time ± SD)	Mortality %
Control	6	0	28	0
0.03	5	0	28	0
0.1	8	3	15, 22, 27, 28, 28, 28, 28, 28 (25.5 ± 4.7)	38
0.3	7	3	17, 18, 24, 28, 28, 28, 28 (24.4 ± 5.0)	43
1	10	6	2, 2, 14, 15, 22, 25, 28, 28, 28, 28 (19.2 ± 10.5)	60
3.5	8	6	0.3, 8, 9, 15, 15, 16, 28, 28 (14.9 ± 9.6)	75
10	3	3	8, 10, 18 (12.0 ± 5.3)	100

**Table 2:** Effect of repeated oral exposure of mice to PLTX on body weight (BW) and accumulated weight change during the treatment period. Values are expressed as mean ± SEM and the number of mice at each week and dose is shown in parenthesis (* *p* < 0.05; ** *p* < 0.01; *** *p* < 0.001 vs. control values).

	Day 0	Day 7	Day 14	Day 21	Day 28
Control	(*n* = 6)	(*n* = 6)	(*n* = 6)	(*n* = 6)	(*n* = 6)
Body weight (g)	22.2 ± 0.9	23.9 ± 0.6	25.9 ± 0.5	27.1 ± 0.8	27.3 ± 0.7
Cumulative BW (g)	0.0	1.7 ± 0.4	3.7 ± 0.6	4.8 ± 0.8	5.1 ± 0.9
0.03 µg/kg	(*n* = 5)	(*n* = 5)	(*n* = 5)	(*n* = 5)	(*n* = 5)
Body weight (g)	22.9 ± 0.6	23.5 ± 0.3	24.5 ± 0.2	25.6 ± 0.4	25.8 ± 0.6
Cumulative BW (g)	0.0	0.6 ± 0.4	1.6 ± 0.7	2.7 ± 0.3	2.9 ± 0.3
0.1 µg/kg	(*n* = 8)	(*n* = 8)	(*n* = 8)	(*n* = 7)	(*n* = 5)
Body weight (g)	23.4 ± 0.8	23.7 ± 0.7	25.0 ± 0.6	25.1 ± 0.8	24.2 ± 0.8 *
Cumulative BW (g)	0.0	0.3 ± 0.6	1.6 ± 0.3	2.2 ± 0.7	1.4 ± 1.6
0.3 µg/kg	(*n* = 7)	(*n* = 7)	(*n* = 7)	(*n* = 5)	(*n* = 4)
Body weight (g)	23.7 ± 0.4	23.3 ± 0.6	24.0 ± 0.8	25.7 ± 0.6	26.1 ± 1.5
Cumulative BW (g)	0.0	−0.4 ± 0.7	0.3 ± 0.9 *	1.9 ± 0.4	2.3 ± 1.0
1 µg/kg	(*n* = 10)	(*n* = 8)	(*n* = 8)	(*n* = 6)	(*n* = 4)
Body weight (g)	24.5 ± 0.8	21.8 ± 1.0	21.1 ± 1.0 **	20.1 ± 0.9 ***	21.8 ± 1.2 **
Cumulative BW (g)	0.0	−2.4 ± 0.8 ***	−3.6 ± 0.7 ***	−4.2 ± 1.2 ***	−2.6 ± 1.4 ***
3.5 µg/kg	(*n* = 8)	(*n* = 7)	(*n* = 5)	(*n* = 2)	(*n* = 2)
Body weight (g)	23.6 ± 0.9	20.4 ± 1.3 *	18.6 ± 2.0 **	20.0 ± 3.8 *	21.8 ± 3.0 *
Cumulative BW (g)	0.0	−3.6 ± 0.7 ***	−5.1 ± 2.1 ***	−3.6 ± 3.5 ***	−1.9 ± 2.7 *
10 µg/kg	(*n* = 3)	(*n* = 3)	(*n* = 1)	(*n* = 1)	
Body weight (g)	22.2 ± 1.5	20.0 ± 0.7	21.5	21.9	
Cumulative BW (g)	0.0	−2.2 ± 1.2 *	−3.4 ***	−3.0 ***	

**Table 3:** Main symptoms after oral administration of PLTX by gavage (animals with symptomatology/total animals for each dose).

Symptoms	0.03 µg/kg	0.1 µg/kg	0.3 µg/kg	1 µg/kg	3.5 µg/kg	10 µg/kg
Lethargy	0/5	1/8	3/7	8/10	6/8	3/3
Piloerection	0/5	3/8	3/7	8/10	7/8	3/3
Dyspnea	0/5	2/8	0/7	6/10	4/8	2/3
Cyanosis	0/5	1/8	0/7	1/10	0/8	0/3
Abdominal pain	0/5	2/8	2/7	4/10	7/8	2/3
Kyphosis	0/5	1/8	2/7	4/10	4/8	3/3
Circling	0/5	0/8	0/7	1/10	2/8	0/3
Ataxia	0/5	0/8	0/7	1/10	2/8	1/3
Vocalization	0/5	0/8	0/7	0/10	2/8	1/3
Facial swelling	0/5	1/8	3/7	3/10	1/8	3/3

**Table 4:** Food intake, faeces and urine production over a 24 h period in control mice and in mice treated with PLTX. Urine production was decreased in mice treated with PLTX at doses of 1 and 3.5 µg/kg, but no significant changes were found mainly due to the limited number of animals.

Group/Analysed Parameters	
Control (*n* = 6)	
Food consumption (g)	4.9 ± 0.6
Faeces (g)	1.3 ± 0.2
Urine (mL)	2.5 ± 1.0
0.03 µg/kg PLTX (*n* = 5)	
Food consumption (g)	4.4 ± 0.3
Faeces (g)	1.0 ± 0.4
Urine (mL)	1.1 ± 0.1
0.1 µg/kg PLTX (*n* = 5)	
Food consumption (g)	4.4 ± 0.2
Faeces (g)	1.3 ± 0.1
Urine (mL)	1.2 ± 0.2
0.3 µg/kg PLTX (*n* = 4)	
Food consumption (g)	3.8 ± 0.6
Faeces (g)	1.5 ± 0.1
Urine (mL)	1.3 ± 0.5
1 µg/kg PLTX (*n* = 4)	
Food consumption (g)	4.3 ± 0.3
Faeces (g)	1.0 ± 0.2
Urine (mL)	0.3 ± 0.1
3.5 µg/kg PLTX (*n* = 2)	
Food consumption (g)	3.4 ± 0.8
Faeces (g)	0.9 ± 0.4
Urine (mL)	0.3 ± 0.2

**Table 5:** Analytical results of urine in control animals and in animals treated daily with PLTX at doses ranging from 0.03 µg/kg to 3.5 µg/kg for 28 days. Values are expressed as mean ± SEM and the number of animals used to evaluate urine parameters is shown in parenthesis for each dose. * *p* < 0.05 versus control animals. For urine protein, glucose, ketones, blood haemoglobin, and bilirubin values represent mean ± SEM (in bold) and after the individual values for each mouse.

Group/Analyzed Parameters	Control (*n* = 6)	0.03 µg/kg (*n* = 5)	0.1 µg/kg (*n* = 5)	0.3 µg/kg (*n* = 4)	1 µg/kg (*n* = 3)	3.5 µg/kg (*n* = 2)
Colour	(2) Pale Yellow (3) Amber (1) Dark Yellow	(1) Pale Yellow (1) Amber (3) Dark Yellow	(3) Amber (2) Dark Yellow	(4) Amber	(3) Amber	(2) Amber
Turbididy	(1) Clear (1) Slightly cloudy (4) Cloudy	(3) Clear (2) Cloudy	(1) Slightly cloudy (4) Cloudy	(4) Very Cloudy	(3) Cloudy	(2) Cloudy
Specific Gravity	(1) 1020 (1) 1032 (4) >1050	(2) 1030 (1) 1032 (2) >1050	(1) 1028 (4) >1050	(1) 1038 (1) 1050 (2) >1050	(3) >1050	(2) >1050
Urine protein (g/L)	0.4 ± 0.2 (3) 0 (1) Trace (2) 1	0 ± 0 (4) 0 (1) Trace	0.4 ± 0.2 (1) 0 (3) 0.3 (1) 1	0.3 ± 0 (1) Trace (3) 0.3	2.1 ± 1.5 (1) 0.3 (1) 1 (1) 5	0.7 ± 0.4 (1) 0.3 (1) 1
Glucose (mmol/L)	1.5 ± 0.7 (3) 0 (3) 3	3 ± 0 (5) 3	5.8 ± 2.8 (4) 3 (1) 17	3 ± 0 (4) 3	0 ± 0 (2) 0	0 ± 0 (2) 0
Ketones (mmol/L)	0.8 ± 0.4 (3) 0 (3) 1.5	0.3 ± 0.3 (4) 0 (1) 1.5	1.2 ± 0.3 (1) 0 (4) 1.5	0.4 ± 0.4 (3) 0 (1) 1.5	0.8 ± 0.8 (1) 0 (1) 1.5	0 ± 0 (2) 0
Blood/Haemoglobin (Ery/µL)	1.6 ± 1.6 (5) 0 (1) 10	0 ± 0 (5) 0	12 ± 9.7 (3) 0 (1) 10 (1) 50	2.5 ± 2.5 (3) 0 (1) 10	0 ± 0 (2) 0	5 ± 5 (1) 0 (1) 10
Bilirubin (µmol/L)	11.2 ± 8.3 (4) 0 (1) 17 (1) 50	10.2 ± 4.2 (2) 0 (3) 17	43.4 ± 6.6 * (1) 17 (4) 50	25.3 ± 8.3 (3) 17 (1) 50	17 ± 0 (2) 17	50 ± 0 * (2) 50
Urobilinogen (µmol/L)	2.8 ± 2.8 (5) 0 (1) 17	10.2 ± 4.2 (2) 0 (3) 17	6.8 ± 4.2 (3) 0 (2) 17	0 ± 0 (4) 0	8.5 ± 8.5 (1) 0 (1) 17	8.5 ± 8.5 (1) 0 (1) 17

## References

[1] Moore R.E., Scheuer P.J. (1971). Palytoxin: A new marine toxin from a coelenterate. Science.

[2] Ciminiello P., Dell’Aversano C., Fattorusso E., Forino M., Magno G.S., Tartaglione L., Grillo C., Melchiorre N. (2006). The Genoa 2005 outbreak. Determination of putative palytoxin in mediterranean *Ostreopsis ovata* by a new liquid chromatography tandem mass spectrometry method. Anal. Chem..

[3] Ciminiello P., Dell’Aversano C., Fattorusso E., Forino M., Tartaglione L., Grillo C., Melchiorre N. (2008). Putative palytoxin and its new analogue, ovatoxin-a, in *Ostreopsis ovata* collected along the Ligurian coasts during the 2006 toxic outbreak. J. Am. Soc. Mass Spectrom..

[4] Onuma Y., Satake M., Ukena T., Roux J., Chanteau S., Rasolofonirina N., Ratsimaloto M., Naoki H., Yasumoto T. (1999). Identification of putative palytoxin as the cause of clupeotoxism. Toxicon.

[5] Nincevic Gladan Z., Arapov J., Casabianca S., Penna A., Honsell G., Brovedani V., Pelin M., Tartaglione L., Sosa S., Dell’Aversano C. (2019). Massive occurrence of the harmful benthic dinoflagellate *Ostreopsis* cf. *ovata* in the eastern Adriatic Sea. Toxins.

[6] Accoroni S., Romagnoli T., Penna A., Capellacci S., Ciminiello P., Dell’Aversano C., Tartaglione L., Abboud-Abi Saab M., Giussani V., Asnaghi V. (2016). Ostreopsis fattorussoi sp. Nov. (dinophyceae), a new benthic toxic Ostreopsis species from the eastern Mediterranean Sea. J. Phycol..

[7] Aligizaki K., Katikou P., Nikolaidis G., Panou A. (2008). First episode of shellfish contamination by palytoxin-like compounds from *Ostreopsis* species (Aegean Sea, Greece). Toxicon.

[8] Amzil Z., Sibat M., Chomerat N., Grossel H., Marco-Miralles F., Lemee R., Nezan E., Sechet V. (2012). Ovatoxin-a and palytoxin accumulation in seafood in relation to *Ostreopsis* cf. ovata blooms on the French Mediterranean coast. Mar. Drugs.

[9] David H., Moita M.T., Laza A., Mateus M., Pablo H., Orive E. (2012). First bloom of *Ostreopsis* cf. *ovata* in the continental Portuguese coast. Harmful Algae News.

[10] Solino L., Garcia-Altares M., Godinho L., Costa P.R. (2020). Toxin profile of Ostreopsis cf. ovata from Portuguese continental coast and Selvagens Islands (Madeira, Portugal). Toxicon.

[11] Accoroni S., Tartaglione L., Dello Iacovo E., Pichierri S., Marini M., Campanelli A., Dell’Aversano C., Totti C. (2017). Influence of environmental factors on the toxin production of *Ostreopsis* cf. ovata during bloom events. Mar. Pollut. Bull..

[12] Botana L.M. (2016). Toxicological perspective on climate change: Aquatic toxins. Chem. Res. Toxicol..

[13] Silva M., Pratheepa V.K., Botana L.M., Vasconcelos V. (2015). Emergent toxins in North Atlantic temperate waters: A challenge for monitoring programs and legislation. Toxins.

[14] Alcala A.C., Alcala L.C., Garth J.S., Yasumura D., Yasumoto T. (1988). Human fatality due to ingestion of the crab Demania reynaudii that contained a palytoxin-like toxin. Toxicon.

[15] Kodama A.M., Hokama Y., Yasumoto T., Fukui M., Manea S.J., Sutherland N. (1989). Clinical and laboratory findings implicating palytoxin as cause of ciguatera poisoning due to *Decapterus macrosoma* (mackerel). Toxicon.

[16] Okano H., Masuoka H., Kamei S., Seko T., Koyabu S., Tsuneoka K., Tamai T., Ueda K., Nakazawa S., Sugawa M. (1998). Rhabdomyolysis and myocardial damage induced by palytoxin, a toxin of blue humphead parrotfish. Intern. Med..

[17] Taniyama S., Mahmud Y., Terada M., Takatani T., Arakawa O., Noguchi T. (2002). Occurrence of a food poisoning incident by palytoxin from a serranid *Epinephelus* sp. In japan. J. Natl. Toxins.

[18] Murphy L.T., Charlton N.P. (2017). Prevalence and characteristics of inhalational and dermal palytoxin exposures reported to the national poison data system in the U.S. Envon. Toxicol. Pharm..

[19] Patocka J., Nepovimova E., Wu Q., Kuca K. (2018). Palytoxin congeners. Arch. Toxicol..

[20] Thakur L.K., Jha K.K. (2016). Palytoxin-induced acute respiratory failure. Respir. Med. Case Rep..

[21] Hoffmann K., Hermanns-Clausen M., Buhl C., Büchler M.W., Schemmer P., Mebs D., Kauferstein S. (2008). A case of palytoxin poisoning due to contact with zoanthid corals through a skin injury. Toxicon.

[22] Tartaglione L., Pelin M., Morpurgo M., Dell’Aversano C., Montenegro J., Sacco G., Sosa S., Reimer J.D., Ciminiello P., Tubaro A. (2016). An aquarium hobbyist poisoning: Identification of new palytoxins in *Palythoa* cf. *toxica* and complete detoxification of the aquarium water by activated carbon. Toxicon.

[23] Artigas P., Gadsby D.C. (2003). Na^+^/K^+^-pump ligands modulate gating of palytoxin-induced ion channels. Proc. Natl. Acad. Sci. USA.

[24] Artigas P., Gadsby D.C. (2003). Ion occlusion/deocclusion partial reactions in individual palytoxin-modified Na/K pumps. Ann. N. Y. Acad. Sci..

[25] Artigas P., Gadsby D.C. (2004). Large diameter of palytoxin-induced na/k pump channels and modulation of palytoxin interaction by Na/K pump ligands. J. Gen. Physiol..

[26] Poli M., Ruiz-Olvera P., Nalca A., Ruiz S., Livingston V., Frick O., Dyer D., Schellhase C., Raymond J., Kulis D. (2018). *,* et al. Toxicity and pathophysiology of palytoxin congeners after intraperitoneal and aerosol administration in rats. Toxicon.

[27] Rouzaire-Dubois B., Dubois J.M. (1990). Characterization of palytoxin-induced channels in mouse neuroblastoma cells. Toxicon.

[28] Del Favero G., Beltramo D., Sciancalepore M., Lorenzon P., Coslovich T., Poli M., Testai E., Sosa S., Tubaro A. (2013). Toxicity of palytoxin after repeated oral exposure in mice and in vitro effects on cardiomyocytes. Toxicon.

[29] Del Favero G., Sosa S., Poli M., Tubaro A., Sbaizero O., Lorenzon P. (2014). In vivo and in vitro effects of 42-hydroxy-palytoxin on mouse skeletal muscle: Structural and functional impairment. Toxicol. Lett..

[30] Fernandez D.A., Louzao M.C., Vilarino N., Espina B., Fraga M., Vieytes M.R., Roman A., Poli M., Botana L.M. (2013). The kinetic, mechanistic and cytomorphological effects of palytoxin in human intestinal cells (Caco-2) explain its lower-than-parenteral oral toxicity. FEBS J..

[31] Pelin M., Sosa S., Pacor S., Tubaro A., Florio C. (2014). The marine toxin palytoxin induces necrotic death in HaCaT cells through a rapid mitochondrial damage. Toxicol. Lett..

[32] Vale C., Alfonso A., Sunol C., Vieytes M.R., Botana L.M. (2006). Modulation of calcium entry and glutamate release in cultured cerebellar granule cells by palytoxin. J. Neurosci. Res..

[33] Vale C., Gomez-Limia B., Vieytes M.R., Botana L.M. (2007). Mitogen-activated protein kinases regulate palytoxin-induced calcium influx and cytotoxicity in cultured neurons. Br. J. Pharm..

[34] Vale-Gonzalez C., Gomez-Limia B., Vieytes M.R., Botana L.M. (2007). Effects of the marine phycotoxin palytoxin on neuronal pH in primary cultures of cerebellar granule cells. J. Neurosci. Res..

[35] Boente-Juncal A., Vale C., Camina M., Cifuentes J.M., Vieytes M.R., Botana L.M. (2020). Reevaluation of the acute toxicity of palytoxin in mice: Determination of lethal dose 50 (LD_50_) and no-observed-adverse-effect level (NOAEL). Toxicon.

[36] Munday R. (2011). Palytoxin toxicology: Animal studies. Toxicon.

[37] Sosa S., Del Favero G., De Bortoli M., Vita F., Soranzo M.R., Beltramo D., Ardizzone M., Tubaro A. (2009). Palytoxin toxicity after acute oral administration in mice. Toxicol. Lett..

[38] Tubaro A., Del Favero G., Beltramo D., Ardizzone M., Forino M., De Bortoli M., Pelin M., Poli M., Bignami G., Ciminiello P. (2011). Acute oral toxicity in mice of a new palytoxin analog: 42-hydroxy-palytoxin. Toxicon.

[39] Pelin M., Brovedani V., Sosa S., Tubaro A. (2016). Palytoxin-containing aquarium soft corals as an emerging sanitary problem. Mar. Drugs.

[40] Tubaro A., del Favero G., Pelin M., Bignami G., Poli M., Botana L.M. (2014). Palytoxin and Analogues: Biological Effects and Detection. Seafood and Freshwater Toxins: Pharmacology, Physiology and Detection.

[41] EFSA (2009). Panel on contaminants in the food chain (CONTAM). Scientific opinion on marine biotoxins in shellfish—Palytoxin group. EFSA J..

[42] Ito E., Yasumoto T. (2009). Toxicological studies on palytoxin and ostreocin-D administered to mice by three different routes. Toxicon.

[43] OECD (2008). Test No. 407: Repeated Dose 28-Day Oral Toxicity Study in Rodents. https://www.oecd-ilibrary.org/docserver/9789264070684-en.pdf?expires=1596012513&id=id&accname=guest&checksum=AA219AD249F8EB6D4AE423559A037054.

[44] Yu S.P., Choi D.W. (2000). Ions, cell volume, and apoptosis. Proc. Natl. Acad. Sci. USA.

[45] Barbany M., Rossell M., Salvador A. (2019). Toxic corneal reaction due to exposure to palytoxin. Arch. Soc. Esp Oftalmol..

[46] Gaudchau A., Pfeiffer N., Gericke A. (2019). Chemical burns caused by crust anemone. Ophthalmologe.

[47] Hall C., Levy D., Sattler S. (2015). A case of palytoxin poisoning in a home aquarium enthusiast and his family. Case Rep. Emerg. Med..

[48] Wieringa A., Bertholee D., ter Horst P., van den Brand I., Haringman J., Ciminiello P. (2014). Respiratory impairment in four patients associated with exposure to palytoxin containing coral. Clin. Toxicol..

[49] Munday R. (2006). Toxicological requirements for risk assessment of shellfish contaminants: A review. Afr. J. Mar. Sci..

[50] Pirahanchi Y., Jessu R., Aeddula N.R. (2020). Physiology, Sodium Potassium Pump (Na^+^ K^+^ Pump).

[51] Yan Y., Shapiro J.I. (2016). The physiological and clinical importance of sodium potassium ATPase in cardiovascular diseases. Curr. Opin. Pharm..

[52] Qiu L.Y., Krieger E., Schaftenaar G., Swarts H.G., Willems P.H., De Pont J.J., Koenderink J.B. (2005). Reconstruction of the complete ouabain-binding pocket of Na,K-ATPase in gastric H,K-ATPase by substitution of only seven amino acids. J. Biol. Chem..

[53] Rossini G.P., Bigiani A. (2011). Palytoxin action on the Na(+),K(+)-ATPase and the disruption of ion equilibria in biological systems. Toxicon.

[54] Karam S.M. (2010). A focus on parietal cells as a renewing cell population. World J. Gastroenterol..

[55] Karam S.M., Forte J.G. (1994). Inhibiting gastric H(+)-K(+)-ATPase activity by omeprazole promotes degeneration and production of parietal cells. Am. J. Physiol..

[56] Cornejo-Pareja I., Clemente-Postigo M., Tinahones F.J. (2019). Metabolic and endocrine consequences of bariatric surgery. Front. Endocrinol. (Lausanne).

[57] Boente-Juncal A., Vale C., Cifuentes M., Otero P., Camina M., Rodriguez-Vieytes M., Botana L.M. (2019). Chronic in vivo effects of repeated exposure to low oral doses of tetrodotoxin: Preliminary evidence of nephrotoxicity and cardiotoxicity. Toxins.

[58] Abal P., Louzao M.C., Vilarino N., Vieytes M.R., Botana L.M. (2019). Acute toxicity assessment: Macroscopic and ultrastructural effects in mice treated with oral tetrodotoxin. Toxins.

